# The Effect of Cognitive-Behavioral Counseling on Lifestyle in Pregnant Women: A Randomized Controlled Clinical Trial

**DOI:** 10.25122/jml-2019-0163

**Published:** 2020

**Authors:** Mahnaz Farhodimoghadam, Sousan Heydarpour, Nader Salari, Nasrin Jaberghaderi

**Affiliations:** 1. Student Research Committee, Kermanshah University of Medical Sciences, Kermanshah, Iran; 2. Department of Midwifery, Faculty of Nursing and Midwifery, Kermanshah University of Medical Sciences, Kermanshah, Iran.; 3. Department of Biostatistics, School of Health, Kermanshah University of Medical Sciences, Kermanshah, Iran; 4. Department of Psychiatry, Faculty of Medicine, Kermanshah University of Medical Sciences, Kermanshah, Iran

**Keywords:** Cognitive-behavioral counseling, lifestyle, pregnant women

## Abstract

The lifestyle of pregnant women has a close relationship with maternal and fetal health. In pregnant women, health-promoting behaviors lead to the promotion of quality of life and improvement of pregnancy outcomes. Therefore, the present study aimed to determine the effect of cognitive-behavioral counseling on pregnant women’s lifestyle.

This randomized controlled clinical trial study was performed in the health centers of Sanandaj, Iran. Seventy pregnant women were randomly assigned to intervention (n = 35) and control (n = 35) groups. The study was completed with 33 pregnant women in each group. In addition to routine pregnancy care, the control group received, the intervention group received 8 consecutive 60-90-minute counseling sessions with a cognitive-behavioral approach weekly. The Walker Health Promotion Lifestyle Questionnaire was completed before, immediately, and one month after the intervention by the participants of control and intervention groups. Data were collected from February until June 2017. Data were analyzed using SPSS version 16.

The mean score of lifestyle in the intervention and control group was 139.78 ± 21.71 and 142.63 ± 19.12 before the intervention, which reached 151 ± 17.72 and 159.14 ± 14.77, respectively, after the intervention. The difference was significant in the intervention group (P = 0.001) but not in the control group (P = 0.619). Also, the mean scores of the two groups were not significantly different before intervention (P = 0.574), but this difference was statistically significant after the intervention (P = 0.029) and one month after the intervention (P = 0.001).

Based on the results of this study, cognitive-behavioral counseling improves the lifestyle of pregnant women. Therefore, it is suggested that this type of counseling be used along with other services to improve the lifestyle of pregnant women in health care centers.

## Introduction

Pregnancy is one of the most important stages of a woman’s life [1]. In pregnant women, health-promoting behaviors reduce diseases and disabilities, improve quality of life, prevent and even improve physical and mental diseases, and pregnancy outcomes [2]. Lifestyle includes the behavior and personal habits of an individual in everyday life that affects the health of individuals, including pregnant women, and contribute to their health promotion [1]. Today, lifestyle is an essential strategy for preventing non-communicable diseases, and unhealthy lifestyle is reported to cause one-third of the worldwide mortality [3]. Changes in people’s lifestyles are associated with increasing their awareness, changing their behavior, and creating a supportive environment for healthy behaviors [4]. One of the goals of the World Health Organization (WHO) is to promote a healthy lifestyle in the society by 2020 [5]. Six elements of a healthy lifestyle include physical activity, stress management, spiritual growth, health responsibility, interpersonal relationships, and nutrition [6]. The best approach to promote pregnant women’s health includes healthy nutrition, stress management, and exercise. Exercise is associated with beneficiary results [7]. It is recommended that pregnant women exercise at least 150 minutes a week with moderate intensity [8]. However, pregnant women’s concerns about exercise during pregnancy lead to a sedentary lifestyle [9]. Nutrition is another important factor that affects maternal and fetal health during pregnancy [10]. Insufficient nutrition during pregnancy can lead to intrauterine growth retardation (IUGR), abortion, preterm birth, and low birth weight [11]. Inappropriate nutrition in the first months of pregnancy alters fetal brain development, leading to fetal abnormalities [12]. Stress management is one of the approaches to promote pregnant women’s health [7]. In Iranian studies, the prevalence of stress during pregnancy is reported to be 64% [13] and pregnancy stress is associated with adverse outcomes such as abortion, stillbirth, hypertension, gestational diabetes, nausea and vomiting, immune system’s suppression and increased prevalence of episiotomy and neonatal infections, preterm labor, IUGR, low birth weight, reduced maternal and infant’s health in the future [14], as well as intolerance of labor pain [15]. Also, maternal stress increases the risk of a child’s mental diseases such as depression, anxiety, and hyperactivity in the future [16]. Pregnant women who are familiar with stress management methods can take care of themselves better and are more energetic in comparison with stressed women and will experience a better pregnancy [17]. Some have considered counseling an essential factor in health promotion and believe that counseling enables several mechanisms in individuals [18]. The study of Streuling found that counseling can influence lifestyle during pregnancy [19]. Cognitive-behavioral counseling is an approach that teaches individuals new ways of thinking and behaving in order to substitute their negative attitudes about themselves, the world, and the future. This kind of counseling is short and addresses the current problems of the individual to remove them. The cognitive-behavioral approach can be used in correcting misdirected interpretations, guiding negative spontaneities, correcting irrational mental schemas and inadequate cognition, and can be used to set up effective coping responses and control negative emotions [20]. Considering the importance of lifestyle promotion behaviors in pregnant women, the importance of counseling and given the fact that searching the databases revealed no explicit study on the effect of cognitive-behavioral counseling on the lifestyle of pregnant women, we aimed to determine the effect of cognitive-behavioral counseling on the lifestyle of pregnant women who referred to the health centers in Sanandaj.

## Material and Methods

### Study Design and Population

**Figure1:**
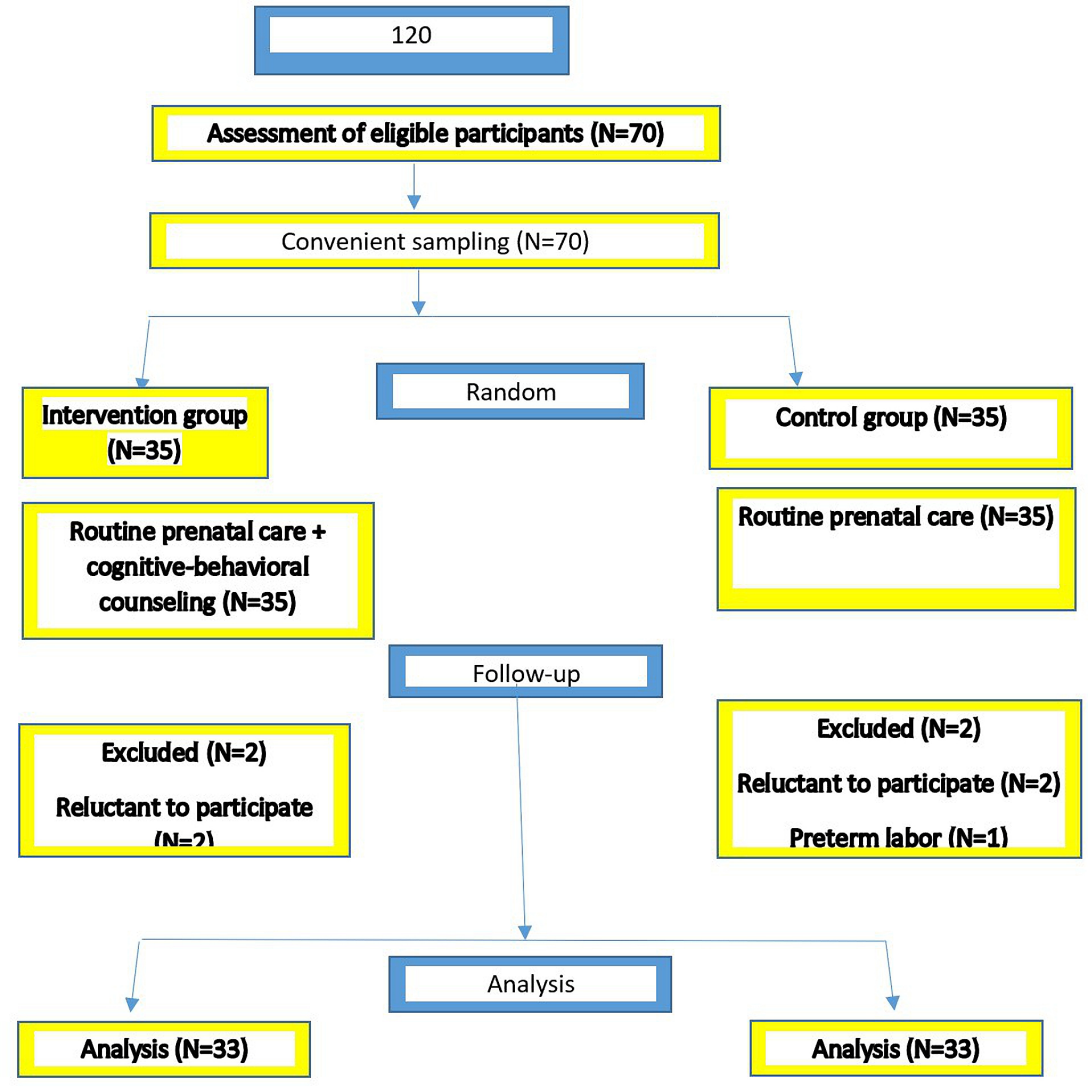
The chart of the study protocol.

In this randomized controlled clinical trial, 70 pregnant women who were referred to Sanandaj health centers were selected continuously and then randomly divided into an intervention and control group (35 individuals each). Finally, 66 pregnant women completed the study, and each group included 33 participants (Figure 1). In the present study, data collection was carried out from February until the end of June 2017. The research environment included the health centers in Sanandaj.

### Methods

The intervention group received eight group-counseling sessions of 60-90 minutes with a cognitive-behavioral approach at weekly intervals conducted by the first author under the supervision of the third author. Counseling was conducted in groups of 11-12 people. The content and structure of the sessions are shown in Table 1. Consultation sessions were held with the coordination of the members of the intervention group at a Healthcare Center in Sanandaj, which was not visited by the members of the control group and had the appropriate equipment and facilities. Both groups received routine pregnancy services and care.

**Table 1:** The content of counselling sessions.

**Session**	**Title**	**Content**	**Presentation method**	**The technique used**
**First**	Introducing the participants and explanation of the program	1. Reviewing the structure of the meetings, rules of the counseling program, and agreeing on the number of sessions, time and place of training and the importance of homework. 2. Familiarity with a cognitive-behavioral approach and group counseling.	- Group discussion - Brainstorming	Techniques: - Verbal promotion - Rewarding
**Second**	Stress management	1. Reviewing the discussion of the previous week and feedback: ten minutes 2. Reviewing and challenging the irrational thoughts and beliefs about stressors. 3. Teaching anger control skills, and time management and strengthening of will. 4. Homework and practical conclusion of the session	- Using a checklist - Group discussion - Slide presentation - Theoretic and practical education - Roleplaying	- Stress management - Deep breathing - Facing emotional and physical moods - Avoiding the stress source - Avoiding catastrophic thinking
**Third**	Exercise	1. Reviewing the last week’s assignments 2. Reviewing and challenging the irrational thoughts and beliefs about exercise during pregnancy and discussion. 3. Training the right exercises during pregnancy. 4. Presenting homework	- Group discussion - Speech - Slide presentation - Practical exercise - Roleplaying - Pamphlet	- Verbal improvement - Problem-solving - Removing obstacles - Activating behavior
**Fourth and fifth**	Nutrition	1. Reviewing the last week’s assignments 2. How to prepare healthy food. 3. Presenting homework	- Group discussion - Collecting thoughts - Speech - Slide presentation	- Identifying self-thoughts - Assessing negative thoughts and replacing them with positive thoughts - Successful experience
**Sixth**	Sleep and relaxation	1. Reviewing the last week’s assignments 2. Familiarity with self-thinking thoughts and cognitive misinterpretations (examination of sleep disorders and insomnia) 3. Teaching methods for stopping thought and returning attention. 4. Presenting homework	- Group discussion - Collecting thoughts - Speech - Slide presentation	- Techniques for dealing with avoidant behavior - Evaluating evidence -Normalization
**Seventh**	Skills for attracting support and presentation	1. Reviewing the last week’s assignments and discussing it: Ten minutes. 2. Examining irrational thoughts and ideas about gaining support and expressing existence. 3. Teaching verbal and nonverbal skills for communicating with others and teaching about the role of interpersonal social relationships in increasing self-control. 4. Presentation of homework, conclusion.	- Group discussion - Speech	- Dealing with avoidant behaviors - Distinction of thought and its understanding - Good, bad, good technique
**Eighth**	Summarizing	1. Reviewing the last week’s assignments 2. Summarizing and reminding what they have learned.	- Group discussion	- Verbal improvement - Rewarding

### Measuring tools

The data collection tool was a socio-demographic questionnaire and descriptive characteristics related to the current pregnancy, completed by the participants of both groups before intervention. Walker’s health promotion lifestyle questionnaire was completed before, immediately and one month after the intervention. Walker et al. reported a Cronbach’s alpha of 94% and test-retest reliability of 89% for the questionnaire (Isazadegan). The validity and reliability of this questionnaire in Iran were confirmed by Cronbach’s alpha for the whole questionnaire at 0.82, and each health dimension from 0.44 to 0.91 [37]. This questionnaire has 52 items and measures health promotion behaviors in six dimensions: nutrition (9 items), physical activity (8 items), health responsibility (9 items), stress management (8 items), interpersonal relationships (9 items), and spiritual growth (9 items). Each question has four choices: never (score 1), sometimes (score 2), often (score 3) and always (score 4); and the final score of each health dimension is obtained by the sum of the total score of questions related to that dimension, and the total score of the questionnaire is obtained by the sum of scores of questions that ranged from 52 to 208.

### Ethical considerations

In order to meet the ethical considerations, the research objectives were first explained to the participants, and they were assured that their information will be kept confidential and that they can leave the study at any time. Ultimately, they signed written informed consent. The present study was confirmed by the Ethics Committee of the Kermanshah University of Medical Sciences and was registered in the Iranian clinical trial registration system by the code IRCT2017041014333N72. In order to comply with research ethics, after the end of the study, a group counseling session was held for willing women of the control group. The content of the sessions included cognitive-behavioral counseling, and its techniques were in accordance with the needs of the intervention group.

### Inclusion and exclusion criteria

The inclusion criteria consisted of age >19, gestational age between 20-24 weeks, an educational level higher than elementary school, intentioned pregnancy, no diseases or complications associated with pregnancy (preeclampsia, gestational diabetes, the birth of an infant with IUGR), singleton pregnancy, no current mental disease or embryonic disease, no history of addiction, no medical history of using narcotics or neurological drugs, and not previous participation in educational and counseling programs. The exclusion criteria consisted of pregnancy loss, delivery, or hospitalization during the study and not attending more than two counseling sessions.

### Data Analysis

Data were analyzed using the SPSS software version 16, and descriptive statistics used included frequency, mean and standard deviation, X2, t-test, independent t-test, or the nonparametric equivalents (Wilcoxon and Mann-Whitney test). The significance level for all tests was less than 0.05.

## Results

The results of this study showed no significant difference between the intervention and control groups in terms of educational level, residence, income, number of pregnancies, maternal occupational status, and body mass index (BMI) (Table 2). Analysis of the results showed that the mean score of lifestyle before and after the intervention (95% confidence interval) was statistically significant in the intervention group (P = 0.001) but not in the control group (P = 0.619). Also, the score of lifestyle subscales after the intervention, except for the subscale of health responsibility, was significant in the intervention group, but none of the subscales were significant before and after the intervention in the control group (Table 3).

**Table 2:** Frequency of the individual characteristics of the pregnant women in the intervention and control groups.

**Variable**		**Intervention group** **N = 33**	**Control group** **N = 33**	**Statistical test used**
**Mother’s age**		26.78±4.91	30.09±6.87	Independent t test, P=0.028
**Age at pregnancy**		22.18±1.44	20.69±1.13	Mann-Whitney P=0.001
**Body mass index**		26.45±3.89	26.51±4.81	Independent t test, P=0.963
**Mother’s education level**	**Under Diploma and Diploma**	24 (72.7)	27 (81.8)	Yates correction P=0.387
	**Academic**	9 (27.3)	6 (18.2)	
**Income level**	**Less than ten million **	20 (60.6)	18 (54.5)	Yates correction P=0.402
	**More than ten million IRR**	13 (39.4)	15 (45.5)	
**Gravidity**	**1**	23 (69.7)	21 (63.6)	Yates correction P=0.397
	**2**	10 (30.3)	12 (36.4)	
**Residence**	**Urban**	28 (84.8)	26 (78.8)	Yates correction P=0.375
	**Rural**	5 (15.2)	7 (21.2)	
**Husband’s educational level**	**Under Diploma and Diploma**	14 (42.4)	19 (42.4)	Yates correction P=0.162
	**Academic**	19 (57.6)	28 (84.8)	
**Husband’s job**	**Employed**	27 (81.8)	28 (84.8)	Yates correction P=0.162
	**Others**	6 (18.2)	5 (15.2)	

**Table 3:** Comparison of the mean and standard deviation of lifestyle score and its subscales before, immediately after and one month after the intervention between the intervention and control groups.

	**Intervention group**				**Control group**			
	**Before**	**After**	**One month later**	**P - value**	**Before**	**After**	**One month later**	**P - value**
**Nutrition**	25.45 ± 3.3	28.18 ± 4.03	28.6 ± 4.31	0.001*	25.21 ± 4.85	24.72 ± 3.85	25.42 ± 4.47	0.809*
**Physical activity**	15.6 ± 4.46	18.9 ± 3.83	20.03 ± 4.29	0.001*	15.57 ± 4.69	16.87 ± 6.12	16.15 ± 5.54	0.405*
**Health responsibility**	27.39 ± 4.71	28.54 ± 4.33	28.93 ± 4.42	0.238*	27.12 ± 4.99	27.6 ± 5.2	27.06 ± 4.7	0.412*
**Stress management**	19.45 ± 3.04	20.75 ± 3.03	22.60 ± 3.24	0.001*	19.27 ± 3.45	19 ± 3.95	18.96 ± 4.24	0.743*
**Interpersonal relationships**	26.48 ± 4.8	25.54 ± 5.46	23.96 ± 4.65	0.012*	26.59 ± 4.97	26.45 ± 5.01	27.51 ± 4.74	0.105*
**Spiritual growth**	26.09 ± 5.39	28.09 ± 4.28	28.45 ± 4.72	0.027*	27.96 ± 4.97	27.63 ± 5.31	27.06 ± 4.46	0.220*
**Overall lifestyle**	139.78 ± 21.76	151 ± 17.72	155.78 ± 17.72	0.001**	142.63 ± 19.1	139.15 ± 24.77	139.3 ± 23.35	0.619 **

Note: * Mann-Whitney

** Independent T-test.

Independent t-tests showed no significant difference before intervention in the mean score of lifestyle between intervention and control groups (P=0.574). However, there was a significant difference between the two groups immediately after the intervention (P=0.029) and one month after intervention (P=0.001). Also, before intervention, mean scores of lifestyle subscales including nutrition (p=0.611), exercise (p=0.099), health responsibility (p=0.820), stress management (p=0.821), interpersonal relationships (p=0.745) and spiritual growth (p=0.146) did not show significant difference between the two groups. However, after the intervention, the mean score of nutrition, exercise, stress management, interpersonal relationships, and spiritual growth subscales was significantly different between two groups, but there was no significant difference between two groups in health responsibility (Table 4).

**Table 4:** Comparison of the mean and standard deviation of total score and subscales of lifestyle between intervention and control groups before, immediately after and one month after the intervention.

	**Intervention group**			**Control group**			**P-value**		
	**Before**	**After**	**One month later**	**Before**	**After**	**One month later**	**Before**	**After**	**One month later**
**Nutrition**	25.45 ± 3.3	28.18 ± 4.03	28.6 ± 4.31	25.21 ± 4.85	24.72 ± 3.85	25.42 ± 4.47	0.611*	0.001**	0.005**
**Physical activity**	15.6 ± 4.46	18.9 ± 3.83	20.03 ± 4.29	15.57 ± 4.69	16.87 ± 6.12	16.15 ± 5.54	0.979**	0.105*	0.002**
**Health responsibility**	27.39 ± 4.71	28.54 ± 4.33	28.93 ± 4.42	27.12 ± 4.99	27.6 ± 5.2	27.06 ± 4.7	0.820**	0.429*	0.099**
**Stress management**	19.45 ± 3.04	20.75 ± 3.03	22.60 ± 3.24	19.27 ± 3.45	19 ± 3.95	18.96 ± 4.24	0.821**	0.047**	0.001**
**Interpersonal relationships**	26.48 ± 4.8	25.54 ± 5.46	23.96 ± 4.65	26.59 ± 4.97	26.45 ± 5.01	27.51 ± 4.74	0.745**	0.585*	0.003*
**Spiritual growth**	26.09 ± 5.39	28.09 ± 4.28	28.45 ± 4.72	27.96 ± 4.97	27.63 ± 5.31	27.06 ± 4.46	0.146**	0.035*	0.020*
**Overall lifestyle**	139.78 ± 21.76	151 ± 17.72	155.78 ± 17.72	142.63 ± 19.1	139.15 ± 24.77	139.3 ± 23.35	0.574**	0.029**	0.001*

Note: * Mann-Whitney

** Independent T-test.

## Discussion

The results of this study showed that holding group counseling sessions with a cognitive-behavioral approach in the intervention group caused a significant change in the mean score of lifestyle in different time intervals (immediately and one month after intervention). In contrast, in the control group, the trend of changes in the mean score of lifestyle was not significant. Immediately and one month after cognitive-behavioral counseling, there was a significant difference in the mean score of lifestyle between the intervention and control groups. Other studies have reported that cognitive-behavioral counseling and treatment improve the quality of life of patients with inflammatory bowel disease [21].

Changes in the lifestyle of patients with type II diabetes [22] also improves the physical and mental health of people with a history of the cardio-metabolic syndrome, primary prevention of type II diabetes mellitus, cardiovascular disease, and at-risk people [24] and improves lifestyle behaviors during pregnancy [25]. In explaining the effect of cognitive-behavioral counseling on the lifestyle of pregnant women, it seems that the techniques trained in this type of counseling help identify unhealthy lifestyle behaviors and then teach strategies to deal with these behaviors. These techniques include a variety of cognitive-behavioral techniques used in this study, such as identifying self-thought techniques, dealing with the incorrect cognition of a disaster, problem-solving, and relaxation techniques, with proven effectiveness in improving lifestyle and modifying unhealthy behaviors [26]. The actual mechanism of the effect of cognitive-behavioral counseling on the lifestyle of pregnant mothers helps them to identify intellectual mistakes, irrational beliefs, change these biased thoughts, and ineffective behavior through systematic discussions and well-organized behavioral assignments [27]. Also, the group nature of the study can be one of the reasons for helping improve lifestyle and modifying health-promoting behaviors in pregnant women because individuals could learn healthy lifestyle behaviors from others and discover that others also have unhealthy behaviors related to lifestyle in pregnancy, such as physical inactivity, incorrect diet, stress and anxiety related to pregnancy and childbirth, and could face different situations in pregnancy through exchanging information and empathy. Based on the results of this study, group counseling with a behavioral approach, dimensions of nutrition, exercise, interpersonal relationships, stress management, and spiritual growth of lifestyle improved. However, in the health responsibility dimension, there was no significant difference between the control and intervention groups. In the study of Pender et al., the lowest score was related to health responsibility as well, which is consistent with the current study [28]. In the study of Radmehr et al., the results showed a significant effect of education based on Pender’s model on the promotion of lifestyle scores in obsessive-compulsive patients but did not affect the subscales of health responsibility and spiritual growth of obsessive patients [29]. In the study of Khazaei et al. on the factors affecting health promotion behaviors among students in Birjand University of Medical Sciences, it has been indicated that the lowest score was in the health responsibility subscale [29]. Wei et al. found the lowest score in health responsibility and physical activity while assessing the health promotion lifestyle of Japanese university students [30]. Regarding the health responsibility and non-significant difference of this subscale in this study, it can be noted that 72.7% of the pregnant mothers of the intervention group had a level of education below diploma and 60.6% of them had an income below 10 million Iranian Rial, which could indicate that a high percentage of pregnant women in the intervention group were from low-income groups and had low health responsibilities due to their economic and social concerns. In general, middle-aged Iranian women have low health responsibilities. Therefore, social media and health workers need to improve the responsibility of pregnant mothers, develop their education on health dimensions, and raise women’s awareness through films, brochures, and pamphlets [31]. The study of Bahar et al. also reported low health responsibilities in Turkish women due to low levels of education, not paying attention to health controls, lack of health insurance, and high cost of health services [32]. Age has been mentioned as an effective factor in health responsibility, and it has been suggested that health responsibility and overall health-promoting behaviors, will increase with age [33]. On the other hand, young people have a good health condition and feel unharmed, which will lead to a lack of adherence to their health-promoting behaviors. In this study, pregnant women in the intervention group were in the young generation of the society with an average age of 28.78 years; this issue may have an impact on their health responsibilities. In the study by Asadnia et al., cognitive-behavioral therapy improved all six subscales of health promotion lifestyle [26]. In other studies, the highest score of health promotion lifestyle subscales was related to the subscales of health responsibility and spiritual growth [34, 35]. In the study by Tal-Azar et al., health responsibility had the highest score [35]. The contradictions in the above studies could be due to cultural differences, differences in target groups, differences in the implementation method of the intervention, and educators’ choice. The strengths of the present research included the random allocation of samples into intervention and control groups, and the limitations of this research included short-term follow-up (one month) and the self-report tool used in this research.

## Conclusion

This study’s results indicated the effectiveness of cognitive-behavioral counseling on improving the lifestyle associated with pregnant mothers’ health. Accordingly, it is suggested that this method be selected and used to promote the lifestyle in women of reproductive age. For this purpose, in order to increase the effectiveness of counseling, in addition to paying attention to cognitive errors and negative self-thoughts, special attention should be paid to the underlying assumptions and incorrect core beliefs of pregnant mothers and correcting them, in order to increase their coping skills in dealing with unhealthy life.

## Acknowledgments

This research is a project of Kermanshah University of Medical Sciences with the code 96077. As a result of this, we thank and appreciate the deputy research chairman of Kermanshah University of Medical Sciences who financially supported the project and the personnel of Sanandaj health centers and the pregnant women participating in this research.

## Conflict of Interest

The authors declare that there is no conflict of interest.

## References

[1] Moshki M, Bahri N, Sadegh Moghadam L (2012). Lifestyle of pregnant women living in Gonabad (Iran). J Res Health.

[2] Norouzi A, Ghofranipour F, Heydarnia A, Tahmasebi R (2010). Determinants of physical activity based on Health Promotion Model (HPM) in diabetic women of Karaj diabetic institute ISMJ.

[3] Maheri AB, Bahrami M-N, Sadeghi R (2013). Health-promoting lifestyle among the students living in dormitories of Tehran University of Medical Sciences, Iran. J Health Dev.

[4] Bohairaee A, Mirgaforvand M (2011). Health promotion from concepts to application.

[5] Naghibi F, Golmakani N, Esmaily H, Moharari F (2013). The relationship between life style and the health related quality of life among the girl students of high schools in Mashhad, 2012-2013. Iran J Obstet Gynecol Infertil.

[6] El Ansari W, Stock C, Phillips C, Mabhala A, Stoate M, Adetunji H (2011). Does the association between depressive symptomatology and physical activity depend on body image perception? A survey of students from seven universities in the UK. Int J Environ Res Public Health.

[7] Abbasi S, Moazami M, Bijeh N, Mirmajidi SR (2015). Investigation of the relationship between physical activity levels, maternal weight (before delivery) and serum cortisol level (during labor) in nulliparous women. Iran J of Obstet Gynecol Infertil.

[8] Department of Health and Human services. 2008. (2008). physical activity guidelines for Americans. http://health.gov//paguidelines.

[9] Noohi E, Nazemzadeh M, Nakhei N (2010). The study of knowledge, attitude and practice of puerperal women about exercise during pregnancy. Iran J Nurs.

[10] Martin-Gronert MS, Ozanne SE (2006). Maternal nutrition during pregnancy and health of the offspring. Portland Press Limited;.

[11] Jalily M, Barati M, Bashirian S (2015). Using social cognitive theory to determine factors predicting nutritional behaviors in pregnant women visiting health centers in Tabriz, Iran. JECH.

[12] Fallah F, Pourabbas A, Delpisheh A, Veisani Y, Shadnoush M (2013). Effects of nutrition education on levels of nutritional awareness of pregnant women in Western Iran. Int J Endocrinol Metab.

[13] Alipour Z, Lamyeian M, Hajizade E (2011). Anxiety in pregnancy: a risk factor for neonatal outcomes. J Urmia Nurs Midwifery Fac April and May.

[14] Divney AA, Sipsma H, Gordon D, Niccolai L, Magriples U, Kershaw T (2012). Depression during pregnancy among young couples: the effect of personal and partner experiences of stressors and the buffering effects of social relationships. J Pediatr Adolesc Gynecol.

[15] Martini J, Knappe S, Beesdo-Baum K, Lieb R, Wittchen H-U (2010). Anxiety disorders before birth and self-perceived distress during pregnancy: associations with maternal depression and obstetric, neonatal and early childhood outcomes. Early Hum Dev.

[16] Vianna P, Bauer ME, Dornfeld D, Chies JAB (2011). Distress conditions during pregnancy may lead to pre-eclampsia by increasing cortisol levels and altering lymphocyte sensitivity to glucocorticoids. Med Hypotheses.

[17] Schetter CD, Tanner L (2012). Anxiety, depression and stress in pregnancy: implications for mothers, children, research, and practice. Curr Opin Psychiatry..

[18] Ford K, Hoyer P, Weglicki L, Kershaw T, Schram C, Jacobson M (2001). Effects of a prenatal care intervention on the self-concept and self-efficacy of adolescent mothers. J Perinat Educ.

[19] Streuling I, Beyerlein A, Rosenfeld E, Hofmann H, Schulz T, Von Kries R (2011). Physical activity and gestational weight gain: a meta-analysis of intervention trials. BJOG.

[20] Mohamadinejad F, Pedramrazi SH, - Aliasgharpour M, Tabari F (2015). Kazemnejad A. Effect of patient education program on self-effcacy in patients with diabetes Iranian Journal of Nursing Research Spring.

[21] Bennebroek E F, Sprangers MA, Sitnikova K, Stokkers PC, Ponsioen CY, Bartelsman JF (2017). Effectiveness of cognitive–behavioral therapy on quality of life, anxiety, and depressive symptoms among patients with inflammatory bowel disease: A multicenter randomized controlled trial. J Consult Clin Psychol.

[22] Welschen LM, van Oppen P, Dekker JM, Bouter LM, Stalman WA, Nijpels G (2007). The effectiveness of adding cognitive behavioural therapy aimed at changing lifestyle to managed diabetes care for patients with type 2 diabetes: design of a randomised controlled trial. BMC Public Health.

[23] Gao H, Li L, Chen L, Yang R, Mei S, Zhang Y (2016). Effects of lifestyle intervention using patient-centered cognitive behavioral therapy among patients with cardio-metabolic syndrome: a randomized, controlled trial. BMC Cardiovasc Disord.

[24] Lakerveld J, Bot SD, Chinapaw MJ, van Tulder MW, van Oppen P, Dekker JM (2008). Primary prevention of diabetes mellitus type 2 and cardiovascular diseases using a cognitive behavior program aimed at lifestyle changes in people at risk: Design of a randomized controlled trial. BMC Endocr Disord.

[25] Aşcı Ö, Rathfisch G (2016). Effect of lifestyle interventions of pregnant women on their dietary habits, lifestyle behaviors, and weight gain: a randomized controlled trial. J Health Popul Nutr.

[26] Esmaili A, Asadnia S, Easazadeh A, Amirsardari L, Issazadeghan A, Ansari B (2013). Evaluation of the Effectiveness of Cognitive Behavioral Therapy on Decreasing Depression Levels And Improving The Lifestyle Of Patients with Type 2 Diabetes. J Urmia Univ Med Sci.

[27] Howton K Cognitive Behaviour Therapy for Psychiatric Problems: A Practical Guide.

[28] Pender NJ, Walker SN, Sechrist KR, Frank-Stromborg M (1990). Predicting healthpromoting lifestyles in the workplace. Nurs Res.

[29] Radmehr M, Ashktorab T, Neisi L (2013). Effect of the educational program based on Pender’s theory on the health promotion in patients with obsessive-compulsive disorder. JNE.

[30] Ueda A, Wei C-N, Minamoto K, Ueda K, Harada K, Fukumoto K (2012). Assessment of health-promoting lifestyle profile in Japanese university students. Environ Health Prev Med.

[31] Enjezab B, Farajzadegan Z, Taleghani F, Aflatoonian A, Morowatisharifabad MA (2012). Health promoting behaviors in a population-based sample of middle-aged women and its relevant factors in Yazd, Iran. Int J Prev Med.

[32] BAHAR Z, Beşer A, ÖZBIÇAKÇI FŞ, ÖZTÜ RK, HANEY M (2013). Health promotion behaviors of Turkish women.

[33] Huang S-L, Li R-H, Tang F-C (2010). Comparing disparities in the health-promoting lifestyles of Taiwanese workers in various occupations. Ind Health.

[34] Abedi P, Jorfi M, Afshari P (2015). Evaluation of the Health Promotion Lifestyle and its Related Factors in Reproductive Aged Women in Ahvaz, Iran. Community Health J.

[35] Tol A, Tavassoli E, Shariferad GR, Shojaeezadeh D (2013). Health-promoting lifestyle and quality of life among undergraduate students at school of health, Isfahan university of medical sciences. J Educ Health Promot.

